# Pectoral Fin of the Megamouth Shark: Skeletal and Muscular Systems, Skin Histology, and Functional Morphology

**DOI:** 10.1371/journal.pone.0086205

**Published:** 2014-01-21

**Authors:** Taketeru Tomita, Sho Tanaka, Keiichi Sato, Kazuhiro Nakaya

**Affiliations:** 1 Hokkaido University Museum, Hakodate, Hokkaido, Japan; 2 Department of Geology, University of California Davis, Davis, California, United States of America; 3 School of Marine Science and Technology, Tokai University, Shimizu, Shizuoka, Japan; 4 Okinawa Churashima Research Center, Okinawa Churashima Foundation, Motobu, Okinawa, Japan; 5 Hokkaido University, Hakodate, Hokkaido, Japan; Ghent University, Belgium

## Abstract

This is the first known report on the skeletal and muscular systems, and the skin histology, of the pectoral fin of the rare planktivorous megamouth shark *Megachasma pelagios*. The pectoral fin is characterized by three features: 1) a large number of segments in the radial cartilages; 2) highly elastic pectoral fin skin; and 3) a vertically-rotated hinge joint at the pectoral fin base. These features suggest that the pectoral fin of the megamouth shark is remarkably flexible and mobile, and that this flexibility and mobility enhance dynamic lift control, thus allowing for stable swimming at slow speeds. The flexibility and mobility of the megamouth shark pectoral fin contrasts with that of fast-swimming sharks, such as *Isurus oxyrhinchus* and *Lamna ditropis*, in which the pectoral fin is stiff and relatively immobile.

## Introduction

The first specimen of a megamouth shark (*Megachasma pelagios*) was captured off Hawaii in 1976. It was described as a new genus and species in its own family within the order Lamniformes, and it became the third known planktivorous shark, along with basking and whale sharks [1]. Subsequent studies have explored the skeletal and muscular system of this rare shark, mainly in the cranial region, to clarify its phylogenetic relationships with other sharks [2], [3] and to investigate its feeding ecology [3], [4], [5].

The present study reports on the gross anatomy of the pectoral fin of the megamouth shark, and the histological features of the pectoral fin skin. The skeletal and muscular systems of pectoral fins have been used for taxonomic investigations in some shark lineages [6], [7], 8,9. However, the anatomy of the pectoral fin has only been examined in 5 out of 15 species of extant lamniforms: *Carcharodon carcharias*, *Carcharias taurus*, *Lamna nasus*, *Mitsukurina owstoni*, and *Pseudocarcharias kamoharai* [10], [11], [12], [13], [14].

In addition to its use in phylogenetic investigations, the pectoral fin anatomy is useful for understanding and predicting the styles of locomotion of fishes whose ecologies are poorly known. Recent applications of a flow visualization technique and high-speed digital imaging have revealed that sharks regulate lift by movements of their pectoral fins [15], [16], which strongly suggests that the mobility of the pectoral fins is closely linked to lift control.

The purpose of the present study was twofold: 1) to describe the skeletal and muscular systems of the megamouth shark pectoral fin; and 2) to discuss the locomotory style of the megamouth shark based on the study of its fin anatomy.

## Materials and Methods

### Specimens Examined

Anatomical and histological features were examined in two specimens. The first specimen is a female with a total length (TL) of 3.7 m and a body weight (BW) of 361 kg (OCA-P 20110301). It was incidentally caught on 9 July 2007 during a purse seine fishing operation off Ibaraki prefecture, Japan. The specimen was kept frozen at Okinawa Churaumi Aquarium (Okinawa, Japan) prior to anatomical analysis. Following external observations, on 2 March 2011, the left pectoral fin and girdle were removed at Okinawa Churaumi Aquarium and fixed in 20% formalin at Tokai University (Shimizu, Japan) for later anatomical examination.

The second specimen is a female of 5.4 m TL that was incidentally caught in a set net off Shizuoka prefecture, Japan, on 24 June 2011 (OCA-P 20111217). This specimen was also kept frozen at Okinawa Churaumi Aquarium prior to anatomical analysis. The specimen was partly dissected (including the ‘peeling’ off its pectoral fin skin) and fixed in 20% formalin at Okinawa Churaumi Aquarium. Since 20 March 2012 the specimen has been exhibited at the Main Rest House, Churaumi Plaza, next to Okinawa Churaumi Aquarium.

All the specimens of fishes analyzed in this study were accidentally caught by fishermen and are housed at Okinawa Churaumi Aquarium. The aquarium issued permits allowing examination of the specimens as part of this study.

### Anatomical Terminology

The anatomical terminology of the endoskeletal and muscular systems follows that of [8].

### Radial Cartilages of the Pectoral Fin

**Figure 1:**
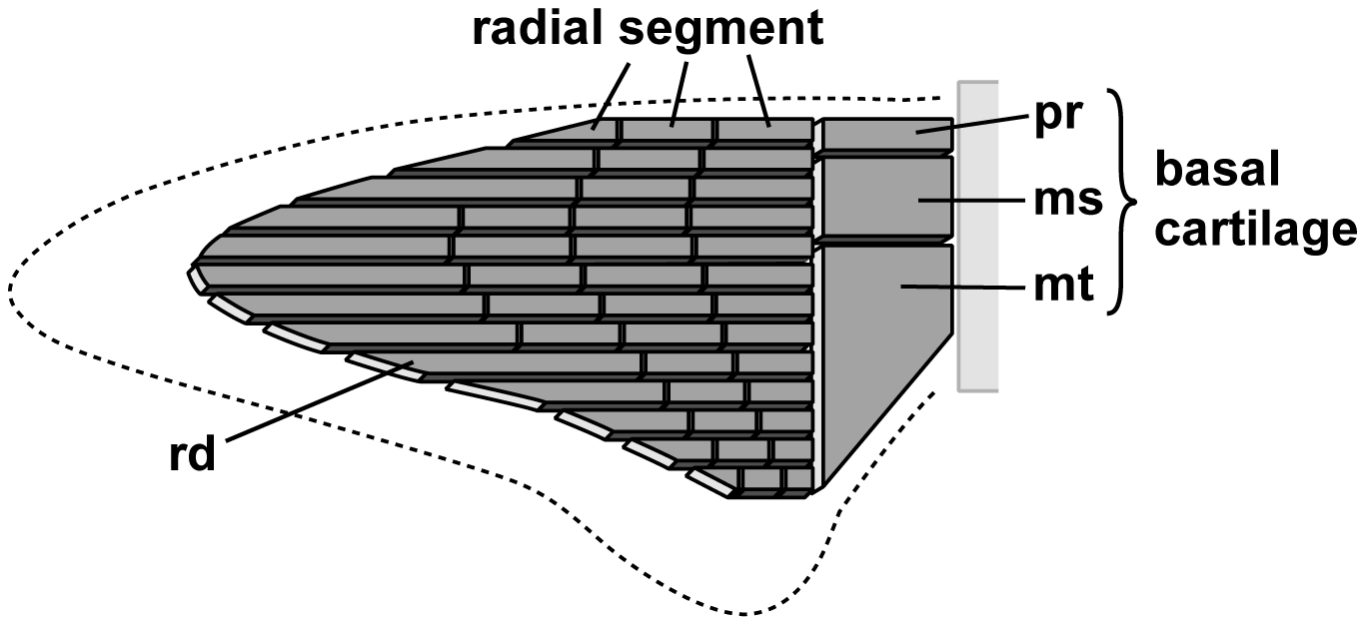
Schematic of the endoskeletal structure of the shark pectoral fin. The radial number and segment number of this pectoral fin are 13 and 4, respectively (see the text for details). ms, mesopterygium; mt, metapterygium; pt, protopterygium; rd, radial.

Elasmobranchs have cartilaginous skeletons whereby individual pieces of the skeleton are comprised of uncalcified cartilage with a mineralized outer layer. The pectoral fins are connected to the pectoral girdle proximally, with the skeletal support for the fins consisting of three larger elements proximally (propterygium, metapterygium, and mesopterygium), articulating distally with a series of radial cartilages (Fig. 1). We measured two variables associated with the radial cartilages of the megamouth shark: 1) the radial number, which is defined as the number of radial cartilages; this number is generally stable during ontogeny and highly uniform within shark species [7]; and 2) the segment number, which is defined as the number of segments in a single radial cartilage. As the pectoral fin consists of multiple cartilages with variable numbers of segments (with the number roughly dependent on the position of the cartilage in the fin), the number of segments in the longest radial cartilage was selected for comparative analysis.

We compared the radial numbers and segment numbers in the megamouth shark with those reported from other lamniform species, including *Alopias vulpinus* [7], *Carcharias taurus* [13], *Carcharodon carcharias* [10], *Cetorhinus maximus* [7], *Lamna nasus* [12], [13], *Mitsukurina owstoni* [11], [13], *Odontaspis ferox* [7], and *Pseudocarcharias kamoharai* [7], [13], [14]. In addition, we examined the pectoral fins of specimens stored at Hokkaido University Museum (Hakodate, Japan), including those of *Alopias superciliosus* (female, 75 cm pre-caudal length, HUMZ133182), *Isurus oxyrinchus* (female, 105 cm TL, HUMZ136626), and *Lamna ditropis* (female, 101 cm TL, HUMZ117738).

### Histology of the Pectoral Fin Skin

**Figure 2:**
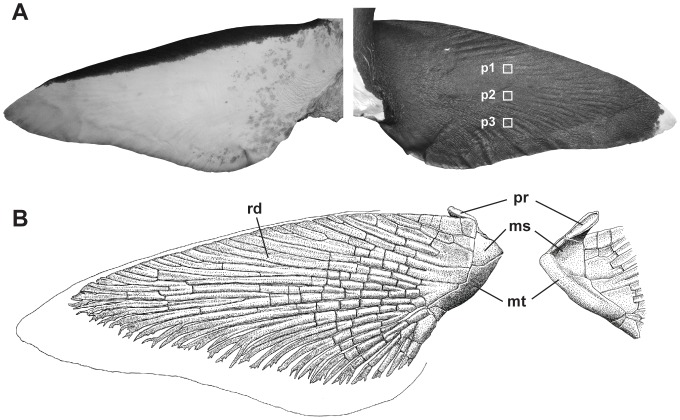
Pectoral fin of the megamouth shark, *Megachasma pelagios* (OCA-P 20110301). (**A**) External morphology of the ventral (left) and dorsal (right) surfaces of the pectoral fin. White boxes represent the sites used for sampling of skin tissues for histological studies. (**B**) Skeletal structure of the ventral side of the pectoral fin (left) and the dorsal side of the basal cartilages (right). ms, mesopterygium; mt, metapterygium; pt, protopterygium; rd, radial.

Skin samples were taken from three sites on the left pectoral fin of megamouth shark specimen OCA-P 20110301 (Fig. 2A). The tissue samples were soaked in 5% formic acid for one week to decalcify the skin denticles, then embedded in paraffin and sectioned transversely (sections of 8–10 µm thickness) using a rotary microtome. The sections were stained with hematoxylin and eosin (HE) for general histological examination, or using the Elastica van Gieson (EVG) staining protocol to examine the extracellular matrixes of the fibrillar connective tissue. In this protocol, the elastic fibers are stained purple-black by the Weigert's resorufin fuchsin solution. The stained specimens were examined using an Olympus light microscope (40–400× magnification), and photographed using an attached digital camera. For comparison, transverse sections of the pectoral fin skin of *Lamna ditropis* (HUMZ117738) and *Isurus oxyrhinchus* (HUMZ136626) were collected and stained using the same methods as those described above.

## Results

### External Morphology

**Figure 3:**
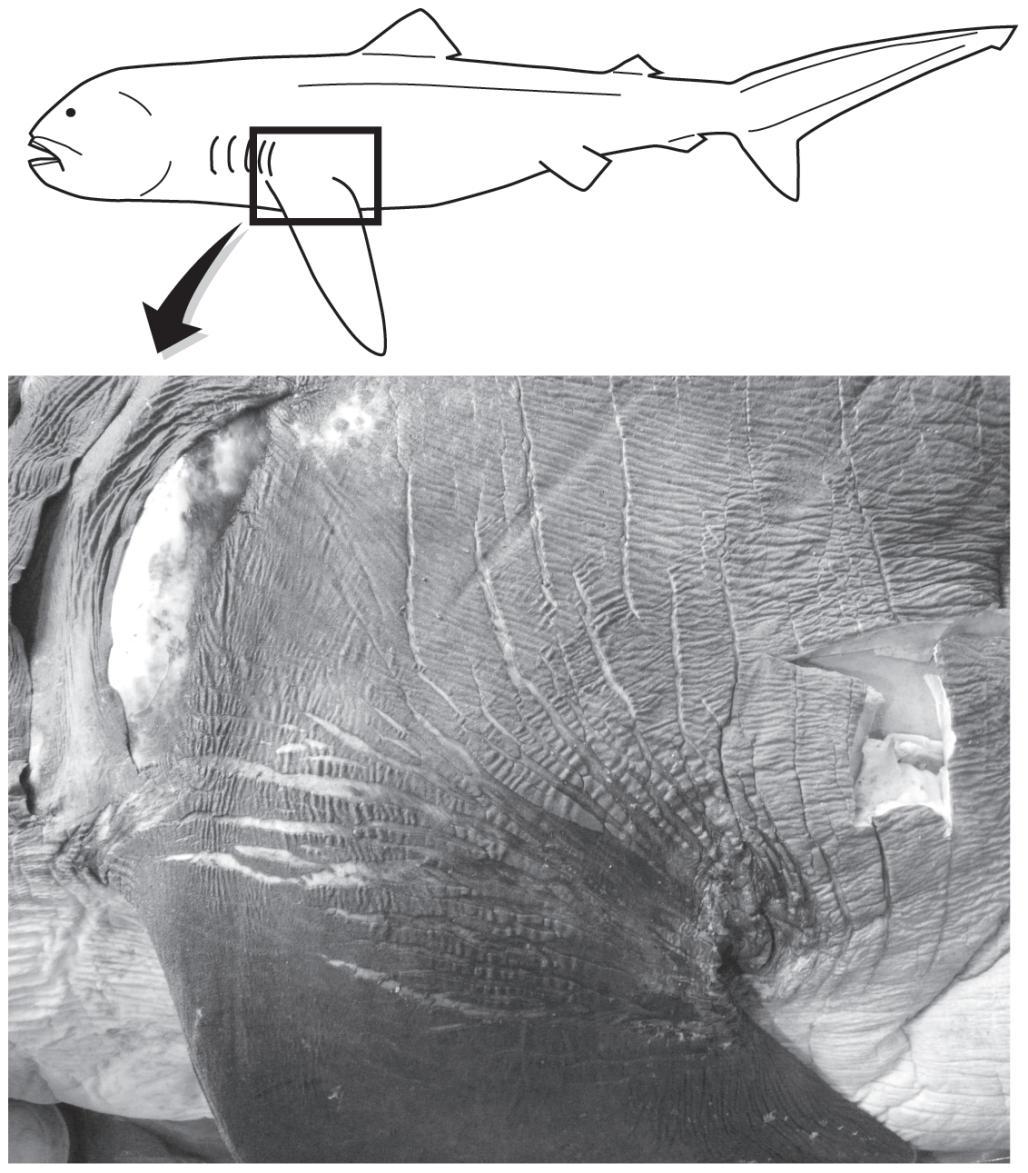
Naked-line networks around the pectoral fin base of the megamouth shark, *Megachasma pelagios* (OCA-P 20111217).

The external morphological measurements of OCA-P 20110301 are presented in Table 1. Pectoral fin is basally broad, distally elongated, tapering, and falcate. Anterior margin is slightly convex. Caudodistal tip is broadly rounded. Posterior margin is nearly straight (Fig. 2A, B). Dorsal surface of pectoral fin is black with a white blotch at the tip. Ventral surface is white with a conspicuously black anterior margin. On both sides of the skin surface of the pectoral fin, there are lines of naked area without dermal denticles, forming a complex network (‘naked-line network’, sensu [17]). The networks, which were previously described from the corner of the mouth and the buccal skin, may function to increase skin elasticity [4], [17]; they are most well developed around the base of the pectoral fin, and the skin around this area is highly stretchable (TT and ST, pers. obs.; also see Fig. 3).

**Table 1:** Pectoral fin measurements of the megamouth shark (OCA-P 20110301; TL = 3.7 m).

Measurement	cm	%TL
Pectoral fin anterior margin	74.9	20.2
Pectoral fin radial length	65.9	17.8
Pectoral fin inner margin	13.8	3.7
Pectoral fin posterior margin	51.7	14.0
Pectoral fin height	66.3	17.9
Pectoral fin length	35.1	9.5

Measurement methods follow those of Compagno (2001).

### Skeletal System

#### Basal cartilages

**Figure 4:**
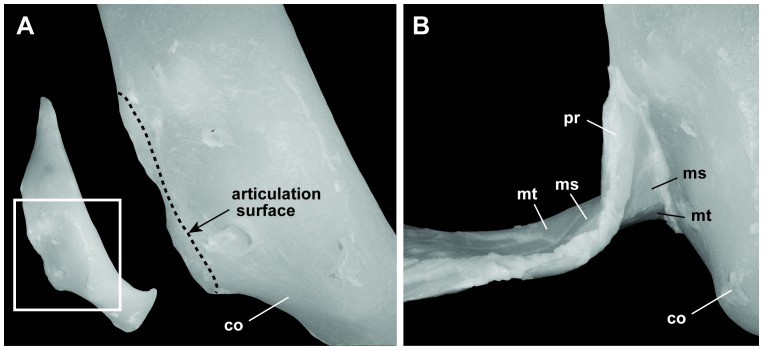
Articulation between the pectoral fin base and the pectoral girdle in the megamouth shark. (**A**) Pectoral girdle: lateral view of the right pectoral girdle (left), and close-up view of the articulation surface for the pectoral fin (right). (**B**) Close-up view of the articulation between the pectoral girdle and the pectoral fin. The pectoral fin base twists approximately 90°. co, coracoid; ms, mesopterygium; mt, metapterygium; pt, protopterygium.

**Figure 5:**
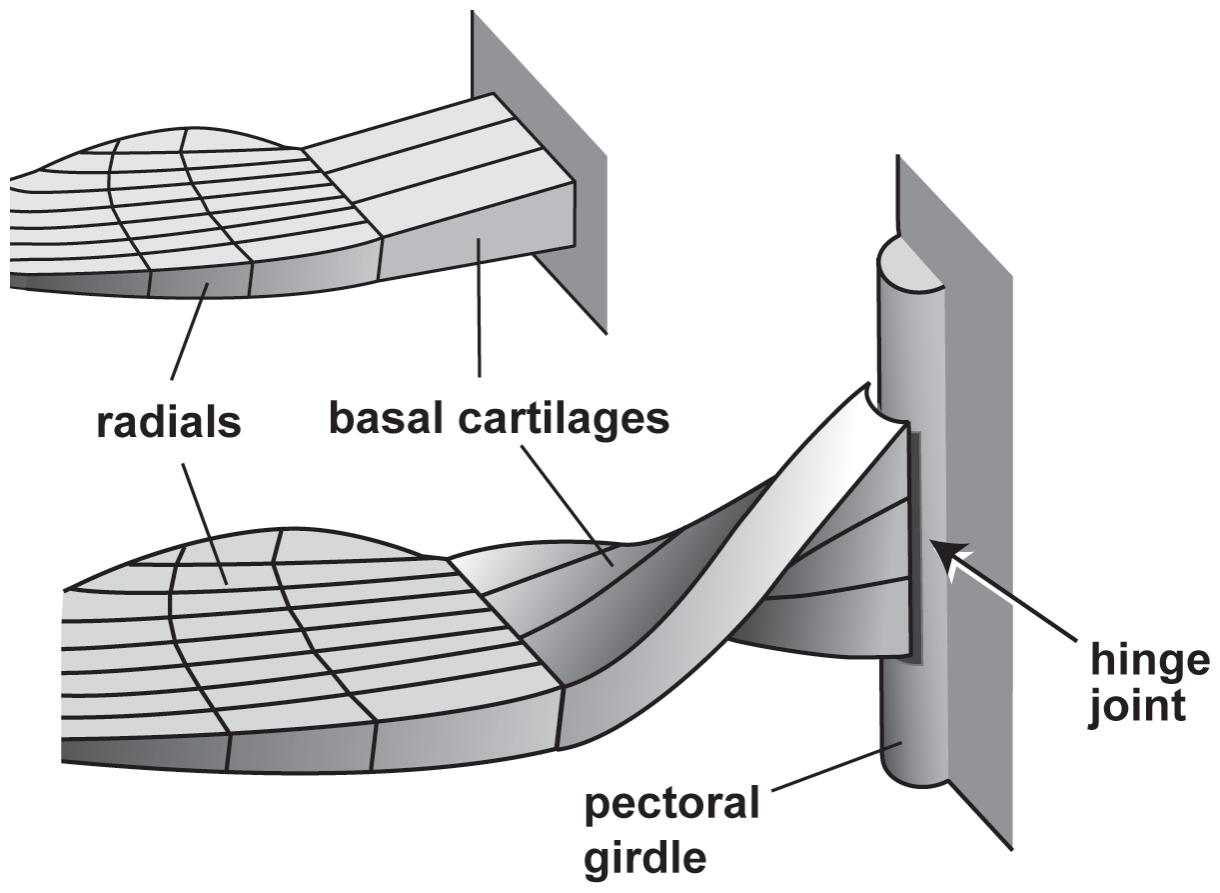
Schematic diagrams of the articulation between the pectoral girdle and the pectoral fin in most sharks (above) and in the megamouth shark (below). In the megamouth shark, the girdle and fin articulate to form a hinge joint in which the rotation axis of the joint is nearly vertical to the plane of the fin.

The proximal region of the pectoral fin consists of three basal cartilages, which, from the anterior to the posterior of the fin, are the propterygium (pt), mesopterygium (ms), and metapterygium (mt) cartilages, as in other sharks (Fig. 2). The basal cartilages are proximally twisted by approximately 90° such that the surfaces that are ventrally directed distally are directed anteriorly at their proximal ends (Fig. 4). The surfaces of the proximal ends of the basal cartilages are smooth, convex, and dorsoventrally elongated; these surfaces fit the articular condyle (Fig. 4A, B) located at the posterior edge of the pectoral girdle, thus forming a hinge joint (Figs. 4B, 5). The rotation axis of the hinge joint is nearly perpendicular to the plane surface of the pectoral fin. The basal cartilages support 23 radials.

#### Radials

Twenty three radial cartilages, which are shaped as slender rods, support the distal region of the pectoral fin. The cartilages, which are oriented towards the anterior pectoral margin, extend 88% of the length of the fin; these features show that the fin is a ‘plesodic-type’ fin (sensu [7]) (Fig. 2). The posterior radial cartilages have 2–6 branches at their distal ends, which distinguishes them from the anterior radial cartilages. Each radial cartilage is segmented into as many as 12 small pieces. Adjacent radial segments belonging to the same radial are articulated with one another by connective tissues.

### Muscular System

**Figure 6:**
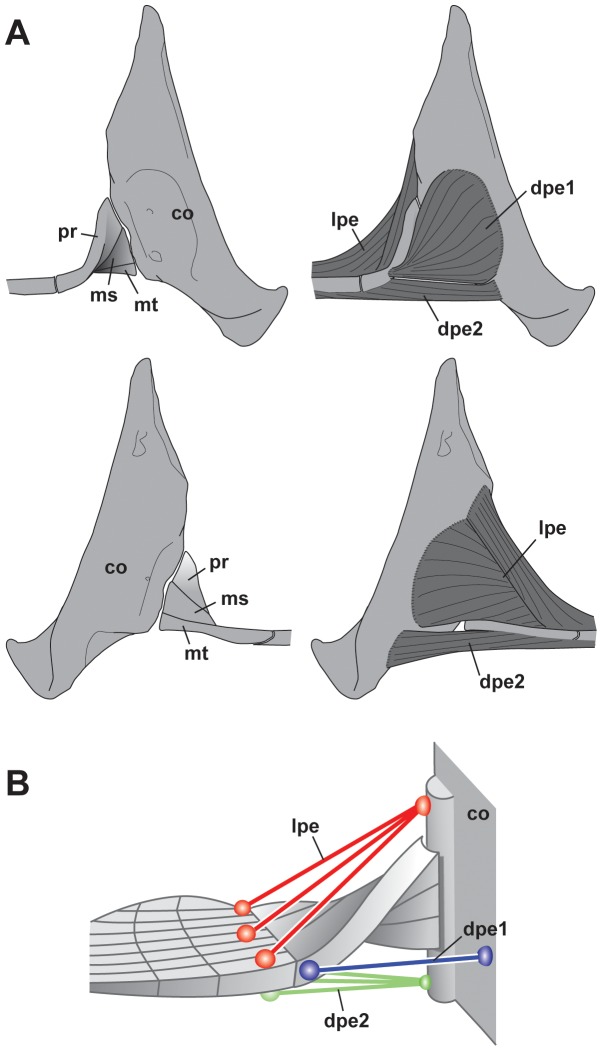
Pectoral muscles of the megamouth shark. (**A**) Lateral view (above) and medial view (below) images. (**B**) Schematic of the pectoral fin muscle systems. co, coracoid; dpe 1, anterior component of the depressor pectoralis; dpe 2, posterior component of the depressor pectoralis; lpe, levator pectoralis; ms, mesopterygium; mt, metapterygium; pt, protopterygium.

The pectoral muscle consists of a dorsal pectoral musculature, the levator pectoralis (lpe), and a ventral pectoral muscle, the depressor pectoralis (dpe) (Fig. 6).

#### 1) Levator pectoralis

The lpe extends posterolaterally and covers the dorsal side of the pectoral fin. It originates from a wide shallow medial concavity in the pectoral girdle, and inserts onto the dorsal sides of the basal cartilages and radials. This muscle extends distally to approximately one-third of the length of the radials.

#### 2) Depressor pectoralis

The dpe is subdivided into two components which have different origins and insertions. The anterior component (dpe 1) originates from a concavity on the lateral side of the pectoral girdle, and inserts on the anterior edge of the propterygium cartilage (pr); this component is referred to as the “protractor muscle” in some references [18], [19]. The posterior component (dpe 2) originates from the ventral side of the pectoral girdle and inserts on the ventral surfaces of the pectoral radials. This muscle extends distally to approximately one-third of the length of the radials. The anterior component (dpe 1) may function to rotate the pectoral fin anteriorly, whereas the posterior component (dpe 2) may function to depress the pectoral fin (Fig. 6B).

### Radial Number and Segment Number

The radial number of the megamouth shark (23) is within the range of the number in other lamniform species (16–34) (Table 2). In contrast, the segment number of the megamouth shark (12) is much larger than that of other lamniform species (2–5).

**Table 2:** Radial number and radial segment number of lamniform sharks.

Species	Radial number	Radial segment number	Reference
*Alopias superciliosus*	34	4	This study^a^
*Alopias vulpinus*	34	n.a.	Compagno, 1988
*Carcharias taurus*	25	4	White, 1937
*Carcharodon carcharias*	28	5	Haswell, 1885
*Cetorhinus maximus*	22	n.a.	Compagno, 1988
*Isurus oxyrinchus*	32	3	This study^b^
*Lamna ditropis*	30	3	This study^c^
*Lamna nasus*	27	3	Garman, 1913; White, 1937
*Megachasma pelagios*	22	12	This study^d^
*Mitsukurina owstoni*	16	2	Jordan, 1898; White, 1937
*Odontaspis ferox*	22	n.a.	Compagno, 1988
*Pseudocarcharias kamoharai*	16	4	Compagno, 1977, 1988

a HUMZ133182,

b HUMZ136626,

c HUMZ117738,

d OCA-P 20110301.

### Skin Histology

**Figure 7:**
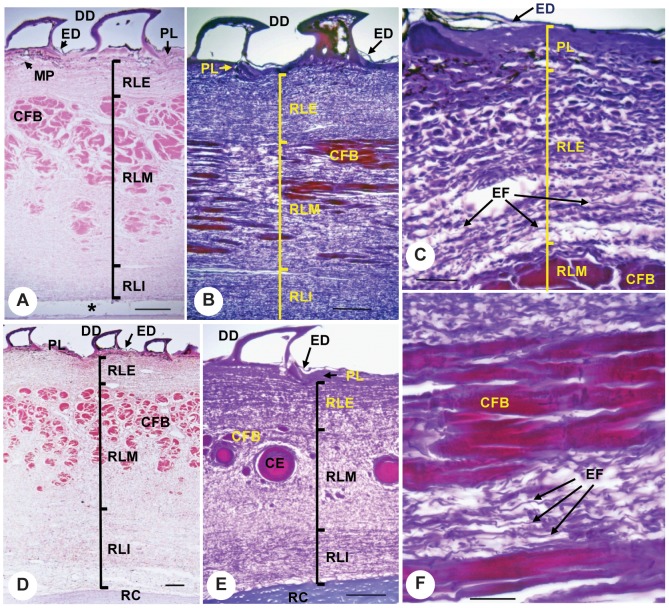
Transverse sections of skin from the dorsal side of the pectoral fin of the megamouth shark. Locations of skin samples (p1, p2, and p3) are shown in Figure 2A. (**A**) Cross-section at site p1 with HE staining. (**B**) Longitudinal section at site p1, with EVG staining. (**C**) Enlarged view of a cross-section of the exterior dermis at site p1, with EVG staining. (**D**) Cross-section at site p2 with HE staining. (**E**) Cross-section at site p3 with EVG staining. (**F**) Enlarged view of a longitudinal section of collagen fiber bundles from site p1 with EVG staining. Scale bars in A, B, D, and E = 0.2 mm, and in C and F = 0.05 mm; ce, ceratotrichia; cfb, collagen fiber bundles; dd, dermal denticles; ed, epidermis; EF, elastic fibers; mp, melanophores; pl, papillary layer; rle, exterior reticular layer; rli, interior reticular layer; rlm, middle reticular layer; rc, radial cartilage; *, artificial spaces produced by the cutting procedure.

The dermis of the megamouth shark consists of papillary and reticular regions (Fig. 7). The former is contiguous with the epidermis and is composed of a thin layer of densely packed cells. The latter mainly consists of an extracellular matrix of collagen and elastic fibers. A high density of elastic fibers was observed in the reticular region, and collagen fiber bundles were abundant only in the middle of this region. The collagen fiber bundles run proximodistally, are gathered together, and vary in size. At site p3, ceratotrichia were observed and the fiber bundles are small and uncommon. The exterior and interior of the reticular region comprise loose connective tissue composed of collagen and elastic fibers.

**Figure 8:**
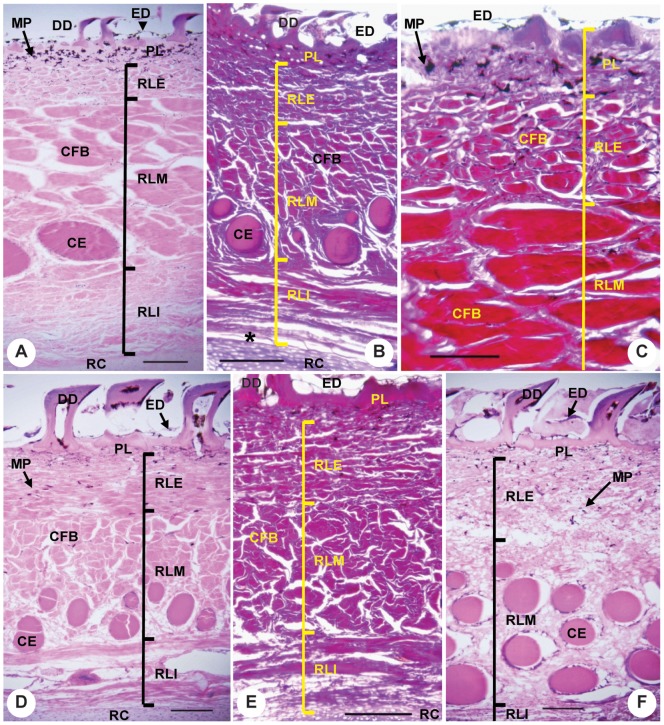
Transverse sections of skin from the dorsal side of the pectoral fin of *Isurus oxyrhinchus* and *Lamna ditropis*. (**A**) Semi-longitudinal section at site p1 of *I. oxyrhinchus* with HE staining. (**B**) Cross-section at site p3 of *I. oxyrhinchus* with EVG staining. (**C**) Enlarged view of a semi-longitudinal section of the exterior dermis at site p1 of *I. oxyrhinchus* with EVG staining. (**D**) Cross-section from site p1 of *L. ditropis* with HE staining. (**E**) Cross-section at site p2 of *L. ditropis* with EVG staining. (**F**) Cross-section at site p3 of *L. ditropis* with HE staining. Scale bar in C = 0.1 mm, and for all others = 0.2 mm.

The dermis of *Lamna ditropis* and *Isurus oxyrhinchus*, as in that of the megamouth shark, consists of papillary and reticular regions (Fig. 8). The papillary region is composed of a thin layer of densely packed cells. The reticular region is composed mainly of dense collagen fiber bundles. Unlike the megamouth shark, there are no elastic fibers in the reticular region. The three layers of the reticular region exhibit distinct angles and sizes of fiber bundles. The exterior layer consists of small fiber bundles, whereas the middle layer comprises large fiber bundles and ceratotrichia. The ceratotrichia are located in the lower part of the layer and are elongate proximodistally at the same angle as the fiber bundles. The fiber bundles of the interior layer are orientated in a different direction to those in the middle layer. At site p3 in *L. ditropis*, the structure of the three layers is obscure, but several ceratotrichia are evident in the interior.

## Discussion

The pectoral fin of the megamouth shark is characterized by the following three features:

1. Large number of radial segments. The number of radial segments in the megamouth shark pectoral fin (12) is so far the greatest among sharks. Because each radial segment in sharks is distally articulated with an adjacent radial segment by connective tissues, the large number of radial segments in the megamouth shark may enhance fin flexibility.
2. High skin elasticity. The skin of the pectoral fin of the megamouth shark is unique in having dense naked-skin networks on its surface. This network may function to increase skin elasticity [4], [17]. The elasticity of the pectoral fin skin is also reflected in the high density of elastic fibers in the reticular region, which is in marked contrast to the abundance of densely packed collagen fiber bundles in *Lamna ditropis* and *Isurus oxyrhinchus*. This suggests that the pectoral fin skin of the megamouth shark is suppler than that of the pectoral fins of the other two sharks.
3. A hinge joint between the pectoral fin and girdle with a rotation axis nearly perpendicular to the fin surface. This structure indicates that the megamouth shark has the capacity for a high degree of rotation of its pectoral fin forward and backward. Such movement is largely restricted in most sharks, where the pectoral fins typically articulate with the coracoid via an anteroposteriorly broad articulation surface, largely limiting pectoral fin motion to the dorsoventral plane (Fig. 5) [8], [20].

Based on these morphological and histological features, we conclude that the pectoral fin of the megamouth shark is highly flexible and mobile. This hypothesis is confirmed by video and still images of a live specimen, in which the pectoral fin is tightly bent, with its ventral side facing upwards (see time segments 00:10–00:22 and 00:36–00:38, in the ARKive video at http://www.arkive.org/megamouth-shark/megachasma-pelagios/video-00.html), and in which the right and left pectoral fins are rotated asymmetrically (ARKive, http://www.arkive.org/megamouth-shark/megachasma-pelagios/image-G5954.html).

The flexible and mobile pectoral fin of the megamouth shark is probably associated with a slow-swimming planktivorous ecology. According to acoustic tracking data, the megamouth shark swims at a speed of 1.5–2.1 km h^−1^ (mean = 1.8 km h^−1^, representing a speed of approximately 0.1 body lengths sec^−1^). This swimming speed is one of the slowest known among elasmobranchs [21]. In general, slow-swimming fishes expend more energy for controlling body posture and depth than do fast-swimming fishes, as locomotory stability is drastically reduced at slow swimming speeds [22]. A flow visualization technique (digital particle image velocimetry, DPIV) and high-speed digital video imaging have shown that sharks actively regulate lift by movements of the posterior portions of the pectoral fins, or by depression/elevation of the pectoral fins [15], [16]. It is therefore likely that the pectoral fins of the megamouth shark are highly specialized for controlling body posture and depth at slow swimming speeds.

The flexibility and mobility of the pectoral fins of the megamouth shark strongly contrast with the stiff and relatively immobile pectoral fins of cruising specialists such as *Isurus oxyrhinchus* and *Lamna ditropis*. Flexible and mobile pectoral fins may provide high controllability for body posture and depth, although the motions cause large hydrodynamic disturbances [22]. The restricted motions of the pectoral fins of *I. oxyrhinchus* and *L. ditropis* may reduce self-generated hydrodynamic disturbances, allowing energetically efficient swimming at high speeds [22], [23].
